# New Pharmacological Insight into Etanercept and Pregabalin in Allodynia and Nociception: Behavioral Studies in a Murine Neuropathic Pain Model

**DOI:** 10.3390/brainsci14111145

**Published:** 2024-11-15

**Authors:** Loulwah Alothman, Emad Alhadlaq, Asma Alhussain, Alwaleed Alabdulkarim, Youssef Sari, Shakir D. AlSharari

**Affiliations:** 1 Department of Oral Medicine and Diagnostic Sciences, College of Dentistry, King Saud University, Riyadh 11451, Saudi Arabia; ehadlaq@ksu.edu.sa (E.A.); dr.asmaalhussain@gmail.com (A.A.); 2 Department of Pharmacology and Toxicology, College of Pharmacy, King Saud University, Riyadh 11451, Saudi Arabiasdalsharari@ksu.edu.sa (S.D.A.); 3 Department of Pharmacology and Experimental Therapeutics, University of Toledo, Toledo, OH 43606, USA

**Keywords:** pregabalin, neuropathic pain, mechanical allodynia, etanercept, tumor necrosis factor-alpha

## Abstract

**Background/Objectives:** Neuropathic pain is challenging to treat, often resistant to current therapies, and associated with significant side effects. Pregabalin, an anticonvulsant that modulates calcium channels, is effective but can impair mental and motor functions, especially in older patients. To improve patient outcomes, reducing the doses of pregabalin and combining it with other drugs targeting different neuropathic pain mechanisms may be beneficial. TNF-α blockers such as etanercept have shown potential in addressing neuropathic pain by affecting sodium channels, synaptic transmission, and neuroinflammation. This study evaluates the efficacy and safety of combining low doses of etanercept and pregabalin in allodynia and nociceptive tests. **Materials and Methods:** Male C57/BL6 mice underwent chronic constriction injury (CCI) of the sciatic nerve to induce neuropathic pain. They were divided into seven groups: sham control, CCI control, low and high doses of pregabalin, low and high doses of etanercept, and a combination of low doses of both drugs. Behavioral tests, including von Frey, hot-plate, and rotarod tests, were used to assess pain responses and motor activity. **Results:** The results indicated that a high dose of pregabalin significantly reduced mechanical allodynia and thermal hyperalgesia but impaired motor function. Conversely, low doses of etanercept alone had no significant effect. However, the combination of low doses of etanercept (20 mg/kg) and pregabalin (5 mg/kg) effectively alleviated pain without compromising locomotor activity. **Conclusions:** These results suggest a novel therapeutic strategy for neuropathic pain, enhancing analgesic efficacy while minimizing adverse effects.

## 1. Introduction

Neuropathic pain is defined by the International Association for the Study of Pain (IASP) as “a pain that arises as a direct consequence of a lesion or disease affecting the somatosensory system” [1,2], including central neurons and peripheral sensory fibers (Aβ, Aδ, and C fibers); these allow for the perception of touch, pain, temperature, pressure, movement, and position via mechanoreceptors, thermoreceptors, chemoreceptors, and nociceptors, which are associated with neuropathic pain and send signals into the spinal cord and the brain. This type of chronic pain is usually long-lasting and can either persist continuously or manifest as recurrent episodes. Neuropathic pain can result from various disorders that affect the peripheral or central nervous system, including metabolic, autoimmune, degenerative, or hereditary diseases, as well as trauma and infection. Additionally, neuropathic pain may arise without any apparent inciting cause, in which case it is termed idiopathic neuropathy [3].

Nociceptive signal-transmitting neurons within the dorsal root ganglion (DRG) contain receptors for proinflammatory cytokines and chemokines, which become upregulated following nerve injury [4,5]. These proinflammatory molecules can sensitize C-fiber nociceptors, resulting in heightened pain sensitivity. Neuroinflammation exacerbates pain hypersensitivity by reducing the activation thresholds of A-δ and C-fiber nociceptors, hence amplifying pain perception. Additionally, nerve injury triggers the infiltration of leukocytes that release high levels of proinflammatory cytokines, which directly sensitize nociceptors and contribute to the development of neuropathic pain [5]. Thus, blocking key proinflammatory cytokines and chemokines can help mitigate neuroinflammation and neuropathic pain. Pregabalin can reduce neuroinflammation during the initial postoperative phase by modulating interactions between peripheral and central neuro-immune systems [6].

The treatment of patients with chronic neuropathic pain is very complicated, and many people do not respond well to the current available medications. Even with the well-known drugs for neuropathic pain, their effectiveness can be unpredictable, dosing can be difficult, the analgesic effects may be delayed, and side effects are common and sometimes serious [7]. Moreover, patients with neuropathic pain often experience significant mental and emotional impairments. Inadequate response to pharmacological therapy is a significant unmet need in patients suffering from neuropathic pain [7,8]. In clinical practice, medications are commonly used to manage neuropathic pain; however, their effectiveness is often inadequate, providing relief to only around 40–50% of patients, and they are frequently associated with undesirable side effects [8]. Pregabalin is a medication with analgesic, antianxiety, and anticonvulsant properties [9]. It is extensively utilized for the management of neuropathic pain, post-herpetic neuralgia, and diabetic neuropathy [7]. Prior research has highlighted the neuroprotective properties of pregabalin in several pain and inflammation models both in vitro and in vivo [10,11,12].

Pregabalin, a well-known analgesic and anticonvulsant, is recognized as a first-line treatment for neuropathic pain according to several international guidelines and FDA approval [7]. Pregabalin is a gamma-amino acid, namely gamma-aminobutyric acid (GABA) carrying an isobutyl substituent at the beta-position (the S-enantiomer). It modulates calcium influx by binding to the alpha-2/delta-1 subunit of the voltage-gated calcium channels in the CNS and the spinal cord [13]. This mechanism of action grants pregabalin its anticonvulsant analgesic and sedative effects. Impairing mental and motor function is one of the most common adverse effects that limit anticonvulsant clinical use, especially in older patients [14]. In an animal model of neuropathic pain, a study by Kato et al. (2017) demonstrated that pregabalin significantly reduced spinal D-serine content and the NMDA/non-NMDA ratio of excitatory postsynaptic currents (EPSCs), thereby attenuating NMDA receptor-mediated synaptic transmission in the spinal cord. This reduction in synaptic transmission is thought to underlie the anti-nociceptive effects of pregabalin in neuropathic pain conditions [15]. Likewise, the α2δ-1 subunit has been shown to be upregulated in dorsal root ganglion (DRG) neurons following injury in neuropathic pain models [16]. Moreover, in vitro investigations (in synaptosomes) have demonstrated that pregabalin diminishes an artificially induced calcium influx and decreases neurotransmitter release within 10–30 min of administration [17]. Additionally, a study by La Porta et al. (2016) showed that pregabalin exhibited a dose-dependent anti-nociceptive effect, proving to be five times more potent than gabapentin. The study further highlighted that pregabalin significantly attenuated mechanical allodynia induced by partial sciatic nerve ligation in mice, as evidenced by a decrease in the frequency of withdrawal responses to mechanical stimuli applied using von Frey filaments, indicating reduced sensitivity to normally non-painful stimuli. Moreover, pregabalin significantly reduced heat hyperalgesia in this neuropathic pain model, as shown by an increase in the latency to respond to noxious thermal stimuli in the hot-plate test, suggesting its effectiveness in alleviating heightened pain sensitivity typically observed in neuropathy [18]. It is important to highlight that pregabalin and gabapentin show efficacy in only 50–60% of patients, and their use is further constrained by a “ceiling” effect as well as significant side effects [19]. As a result, there is a need for better treatment options for managing neuropathic pain in humans. Achieving the effective management of chronic pain through monotherapy is often challenging. Consequently, the use of combination analgesic therapies is considered a rational strategy for managing chronic neuropathic pain [19]. The principle behind multimodal analgesia is to maximize analgesic efficacy (supra-additive synergism) while minimizing adverse effects. This synergistic effect is typically attained by influencing distinct pain pathways or by using a combination of drugs that have complementary mechanisms of action [20].

Combining pregabalin with other agents has been explored to enhance its analgesic effects while minimizing side effects. A study by Lim et al. (2013) demonstrated that the intrathecal administration of ketamine and pregabalin at sub-effective doses in a neuropathic pain model resulted in a synergistic reduction in mechanical allodynia and thermal hyperalgesia without causing motor dysfunction, a common side effect associated with higher doses of pregabalin alone. This suggests that combining treatments at lower doses can enhance pain relief while minimizing adverse effects, such as motor impairment, presenting a promising strategy for effective neuropathic pain management [21]. Moreover, the combination of pregabalin and tolperisone showed a significant anti-allodynic effect, effectively alleviating tactile allodynia in rats induced by partial sciatic nerve ligation (pSNL). This effect was observed within 120 min of acute administration [22].

Another promising modality for neuropathic pain treatment involves TNF-α blockers, such as infliximab, etanercept, and adalimumab [23]. Clinical trials have shown promising results for the efficacy of TNF-α blockers in patients with neuropathic pain. TNF-α is a key mediator in neuropathic pain, influencing voltage-gated sodium channels (VGSCs), affecting synaptic transmissions, and initiating a positive feedback loop with microglial activation, leading to neuroinflammation and associated pain responses [24,25,26]. Etanercept, which consists of two p75 TNF receptors fused to the Fc portion of human IgG [27], has shown significant anti-nociceptive effects in animal models of neuropathic pain. Studies have demonstrated that etanercept, administered either locally or systemically, significantly reduces both mechanical allodynia and thermal hyperalgesia in chronic constriction injury (CCI) models [28,29]. Furthermore, Zanella et al. (2008) demonstrated that a low, locally delivered dose of etanercept using a polymer–drug depot near the injured sciatic nerve provides a prolonged reduction in thermal hyperalgesia [30]. Additionally, gabapentinoids, including pregabalin, are thought to modulate inflammatory processes. For instance, gabapentin might reduce proinflammatory cytokine levels [10], a response linked to an elevated expression of interleukin-10 (IL-10), which is an anti-inflammatory cytokine [31]. Moreover, Kremer et al. (2016) found that while a high dose of pregabalin administered acutely produces a brief anti-allodynic effect, extended treatment with pregabalin is effective in reducing the overproduction of tumor necrosis factor-alpha (TNF-α) triggered by sciatic nerve constriction in the lumbar dorsal root ganglia [31].

To our knowledge, there are no reports on the combination of gabapentinoids and TNF-α blockers for pain management. Given the complementary mechanisms of pregabalin and etanercept—where pregabalin modulates ion channels and etanercept inhibits inflammatory pathways—this study aimed to determine the potential anti-allodynic and anti-nociceptive effects of their combined use in chronic constriction injury (CCI) in mice. By investigating the impact of low-dose combination treatment on various nociceptive tests, this approach could enhance analgesic effects while minimizing side effects, potentially providing a more effective and safer therapeutic strategy for managing neuropathic pain.

## 2. Materials and Methods

### 2.1. Experimental Animals

Experiments were conducted on male C57BL/6J (B6) mice aged 8–10 weeks, weighing approximately 25–30 g. C57BL/6J (B6, Stock No. 000664) mice were obtained from Jackson Laboratories (Bar Harbor, ME, USA). For all studies, the mice were housed at three to five per cage and maintained in our colony at the Animal Center at Pharmacy College, King Saud University. The mice were kept in an accredited animal care facility with a stable temperature of 21 ± 2 °C, humidity levels ranging from 40% to 60%, and a 12 h light/dark cycle, with lights turning on at 7:00 a.m. They were provided unrestricted access to both food and water and each cage contained about 3–4 animals. All the animals included in the study underwent baseline testing prior to any treatment or intervention to ensure consistent baseline measurements across groups. Only healthy animals weighing 25–30 g, displaying normal activity in the locomotor test, successfully trained on the rotarod, responding within the normal range on behavioral tests, and showing no signs of motor dysfunction were selected for inclusion in the study. Prior to initiating the experiments, the mice were acclimated for 1 h. All experiments were conducted during the light phase, specifically between 8:00 a.m. and 5:00 p.m. The research complied with NIH guidelines for the care and use of laboratory animals and obtained approval from the Institutional Animal Care and Use Committee (IACUC) at King Saud University, Saudi Arabia. All experimental procedures conducted on the animals adhered to the guidelines at King Saud University (KSU). Approval for the study was obtained under registration number KSU-SE-22-57.

### 2.2. The Induction of Neuropathic Pain by Utilizing the Chronic Constriction of the Sciatic Nerve (CCI)

A CCI neuropathic pain model was used, which induced the partial denervation of the sciatic nerve through chronic constriction injury (CCI), as described by Bennett and Xie [32,33]. The mice were anesthetized with pentobarbital (45 mg/kg, i.p.). Anesthesia was verified by observing the loss of the righting reflex, ensuring adequate anesthetic depth before proceeding with surgery [34]. Once anesthesia was confirmed, the surgical area was shaved using electric clippers (Wella Professionals, Xpert HS71, Darmstadt, Germany) and subsequently sterilized with Betadine and an alcohol wipe. The eyes were lubricated, and the mice were positioned on a heating pad to provide stable body temperature throughout the surgery. Through a mid-thigh incision, the left common sciatic nerve was exposed. Three ligatures (4/0 prolene) were tied loosely around the nerve just distal to the sciatic trifurcation to ensure the nerve was free of adherent tissue. A distance of 1.0–1.5 mm was kept between each of the three ligatures. Then, the incision was closed with a prolene suture. The ligatures barely constricted the nerve, and the circulation through the superficial epineurial vasculature was not arrested; a slight possibility of nerve movement was allowed [35]. The shaved skin was closed using a suture.

### 2.3. Experimental Grouping and Drug Administration

To evaluate the anti-allodynic and anti-nociceptive effects of pregabalin and etanercept on CCI mice, in total, 56 mice in total were equally divided into seven groups of 8 mice per group: sham control, CCI control, and treatment groups, consisting of high and low doses of pregabalin, high and low doses of etanercept, and the combination treatment group. Pregabalin (unformulated compound) was obtained from Sequoia Research Products Ltd. (Pangbourne, UK) and etanercept (a solution in a prefilled pen) was purchased from Pifzer manufacturing (Betriebsstätte, Freiburg, Germany). The drugs were dissolved in physiological saline (0.9% sodium chloride) and administered intraperitoneally (i.p.) starting on day 4 after CCI surgery, with injections given once daily for 4 days. Pregabalin was administered at doses of 5 and 30 mg/kg, and etanercept at doses of 20 and 40 mg/kg. Doses were selected based on prior studies that have demonstrated pharmacological relevance and safety. The etanercept doses were chosen based on studies showing the effective inhibition of TNF-mediated inflammation in animal models [29,36], while the pregabalin doses were selected according to research on its analgesic efficacy and pharmacodynamic profile [37,38].

**Figure 1:**
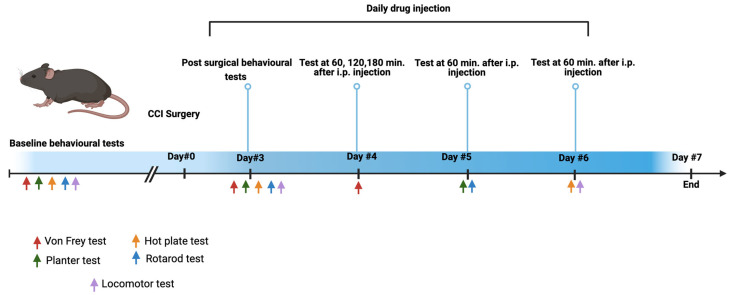
The flowchart of the study design. Baseline pain-related behaviors and motor co-ordination tests were performed on each study group. Neuropathic pain was induced using the chronic constriction of the sciatic nerve (CCI) technique. On day 3 post-surgery, pain-related behavior and motor co-ordination tests were conducted. On day 4 post-surgery, tactile allodynia was assessed using the von Frey test at 60, 120, and 180 min following the intraperitoneal (i.p.) administration of low and high doses of pregabalin, low and high doses of etanercept, a combination of low doses of pregabalin and etanercept, and saline (control). On day 5 post-surgery, the plantar thermal stimulation test and rotarod test were performed 60 min after drug and saline administration. On day 6 post-surgery, both the hot-plate test and locomotor test were conducted 60 min after drug and saline administration. Figure created in BioRender. Alothman, L. (2024) https://BioRender.com/n71a150 (accessed on 31 October 2024).

The sham control and CCI control mice were treated with saline. The evaluation of the pain level was obtained by using three tests: mechanical allodynia, thermal allodynia, and mechanical hyperalgesia (Figure 1). In addition, side effects were assessed by using motor co-ordination tests and locomotor activity tests. Drug administration was performed intraperitoneally at a volume of 0.1 mL per 10 g (10 mg/kg) of mouse body weight using two doses (high and low) and a combination of the low doses of pregabalin and etanercept. Pain behavioral tests and side effects were assessed 60 min after each injection on days 4, 5, and 6 post-CCI surgery.

### 2.4. Behavioral Tests

#### 2.4.1. Von Frey Test (Evaluation of Mechanical Allodynia)

Behavioral assessments were performed on the ipsilateral hind paw of the animals both prior to and following CCI surgery, specifically on postoperative day 4, at different time points, 60, 120, and 180 min after drug administration. Mechanical allodynia was conducted following the procedure outlined by Chaplan et al. [39] using von Frey filaments. The mice were housed in a Plexiglas chamber with a mesh metal floor and were given 1 h to acclimate before the start of testing. By using a modified up-down method, a set of von Frey filaments (Stoelting, Wood Dale, IL, USA) with stiffness levels increasing logarithmically from 3.84 to 5.88, expressing dsLog10 of [10£ force in (mg)], were applied to the paw. If the initially chosen filament did not elicit a withdrawal response, a thicker filament, indicating a stronger stimulus, was used. On the other hand, if a withdrawal response was observed, a weaker filament was introduced next. The force of the filament used first was 0.6 g. Each filament was applied perpendicularly to the paw with enough pressure to create a slight bend, maintaining contact for 2–3 s. The mechanical threshold was expressed as grams, indicating the force of the von Frey hair to which the animal reacted and showing the force of the von Frey hair that elicited a positive reaction from the animal (paw withdrawal, licking, or shaking) [40,41]. Each filament was applied to the paw five times, and the paw withdrawal threshold (PWT) was determined by the smaller of two consecutive filaments that elicited three or more withdrawal responses out of the five attempts [42,43,44]. The experimenter who conducted the tests was blinded to the treatments.

#### 2.4.2. Hot-Plate Test

The mice were introduced into a 10 cm wide glass cylinder placed on a heated plate (Thermojust Apparatus, Columbus, OH, USA) to assess anti-nociception based on the method previously described [45]. The heated plate, surrounded by Plexiglas, maintained a temperature of 55 °C. A manually controlled timer linked to the apparatus measured how long the mouse stayed on the heated surface before showing signs of nociception, such as paw licking or jumping. For each mouse, three control measurements were taken, with intervals of at least 10 min between them. Baseline latency (reaction time), ranging from 8 to 15 s, was set following a saline injection. The heated plate was set to automatically turn off after 40 s to prevent any tissue damage [46]. Each dose and treatment condition involved groups of eight mice. The response time was recorded when the animal exhibited jumping or paw-licking behavior [47,48].

#### 2.4.3. Hargreaves Plantar Test (Evaluation of Thermal Hyperalgesia)

Thermal hyperalgesia was assessed using the Hargreaves Apparatus (Ugo Basile, Varese, Italy) [33,35,49]. The mice were placed in a Plexiglas chamber with a transparent glass base. An infrared beam served as the heat source, targeting the hind paw. The time taken until the first instance of paw withdrawal or licking in reaction to the heat stimulus was recorded as the pain threshold index. Withdrawal latencies were averaged from a minimum of three trials conducted with a 5 min interval between each to obtain the paw withdrawal latency, with a cutoff time of 20 s to prevent tissue damage [33].

#### 2.4.4. Locomotor Activity Test

The measurement of locomotor activity (LA) was conducted based on the York et al. (2013) method [50,51]. LA was assessed using a Supermex apparatus (Muromachi Kikai, Tokyo, Japan) equipped with a sensor monitor positioned above the testing chamber. The open-field area of the chamber measured 25 × 45 × 20 cm^3^. The mice were allowed to habituate in the behavioral testing room for an hour of an acclimation period prior to each experiment in behavioral assays. LA was recorded at three time points: before the start of the chronic constriction injury (CCI) surgery, on day 4 post-surgery before treatment, and on day 6 post-surgery after 60 min of drug administration. Each mouse underwent a 10 min test session upon placement in the testing chamber. Exploratory activity quantified in counts per 10 min was automatically totaled during the test session. An infrared sensor system (SuperMex, Muromachi Kikai, Tokyo, Japan) continuously monitored LA over the 10 min duration. The infrared sensor, positioned centrally on the cage cover with a hole for motion detection, detected the mice’s spontaneous locomotor movements as they freely roamed the open-field area. The data were collected in 1 min blocks over the 10 min test period and presented as the mean ± standard deviation (SD) of changes over time, along with the cumulative activity counts for 10 min. A data analysis was performed using software version 3 (Comp ACT AMS, Muromachi Kikai, Tokyo, Japan).

#### 2.4.5. Motor Co-Ordination Assessment (Rotarod Test)

Motor co-ordination in mice was evaluated using the rotarod test [52,53]. Mice were tested on an automated, accelerating rotarod apparatus (Panlab Harvard Apparatus, LE8200, Barcelona, Spain). Before testing, they were given at least 2 h to acclimate to the testing environment, and the assessments took place between 8:00 a.m. and 1:00 p.m. Only mice pre-trained on the RR at 4 and 10 rpm to maintain balance on the rotating cylinder were selected for the study. Each session included two trials lasting 120 s, with the apparatus sanitized using 70% ethanol between trials. On test day, the rod was set to rotate at a constant speed of 10 rpm with a cutoff time of 120 s. Each mouse was gently held by the tail and positioned on the rod, facing against the rotation. The time each mouse remained on the rod before falling (latency to fall) was recorded in seconds. For each mouse, the average latency was calculated from three trials [50].

### 2.5. Statistical Analysis

The statistical analysis was conducted as follows: Initially, a power analysis calculation was performed using the Lamorte Power Calculator (Boston University Research Compliance, Boston, MA, USA) based on the method by Charan and Kantharia [54]. Based on the power analysis, we chose a sample size of at least eight animals per group to achieve 80% power and detect a mean difference with a significance level of 5%. For the assessment of nociceptive behavior, the calculations indicated that a sample size of five was needed; however, to ensure robustness, we utilized eight mice per group for the nociceptive assay.

The data analysis was conducted utilizing GraphPad Prism software, version 10 (GraphPad Software, Inc., La Jolla, CA, USA). The data are expressed as the mean ± standard deviation (SD). One-way and two-way analysis of variance (ANOVA) tests were conducted, and the Tukey post hoc test was used to evaluate intergroup differences. Statistical significance was defined as *p* < 0.05. All data are expressed as means ± SD.

## 3. Results

### 3.1. Co-Administration of Low Doses of Etanercept and Pregabalin Attenuated Mechanical Allodynia in CCI Mice

**Figure 2:**
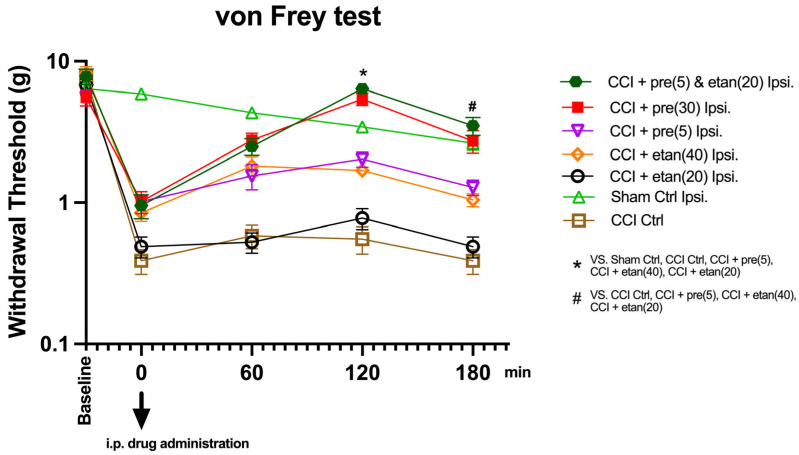
Mean withdrawal thresholds in the von Frey test for postoperative day 4, at different time points (60, 120, and 180 min after i.p. drug administration) among seven groups: sham control, CCI control, pregabalin at 5 mg/kg, pregabalin at 30 mg/kg, etanercept at 20 mg/kg, etanercept at 40 mg/kg, and combination treatment of low doses of pregabalin and etanercept. The data are expressed as the mean ± SD (*n* = 8 per group). Statistical significance was assessed using two-way ANOVA followed by Tukey’s post hoc test. * Statistically significant (*p* < 0.05) for groups “combination treatment” and “pregabalin at 30 mg/kg” (filled legends) compared to other groups at time 120 min. # Statistically significant (*p* < 0.05) for the groups of combination treatment, pregabalin at 30 mg/kg (filled legends), and the sham control compared to other groups at time 180 min.

The mean values of the withdrawal threshold (in grams) at each time point (baseline, 0 min (after CCI and before treatment), 60 min, 120 min, and 180 min) between the seven groups in the von Frey test were compared using a two-way repeated measures analysis of variance (Figure 2). The analysis indicates a highly statistically significant difference in the mean values of withdrawal across the five time points [F (1, 49) = 205.92, *p* < 0.0001]. The interaction term of the time points and groups also shows a highly statistically significant difference in the mean values for withdrawal [F (1, 8) = 10.97, *p* < 0.0001]. Figure 2 shows the comparison of mean values for withdrawal among the seven study groups at each of the five time points, where there is no statistically significant difference among the seven study groups at baseline (*p* = 0.093); the CCI mice displayed a high statistically significant difference with a low withdrawal threshold compared to the sham control mice at all time points (*p* < 0.0001). Furthermore, Figure 2 shows the multiple comparisons of withdrawal mean values among the pairs of the seven study groups at the 0 min time point, showing that the sham control group withdrawal mean values are significantly higher than the mean values of all the other six study groups (*p* < 0.0001). No statistically significant difference in the mean values of the pairs of the other six study groups at the 0 min time point was observed. The multiple comparisons of the withdrawal mean values among the pairs of the seven study groups at 60 min show that the mean values of the sham control group are significantly higher than the mean values of the six groups (CCI control, etanercept at 40 mg/kg, etanercept at 20 mg/kg, pregabalin at 30, pregabalin at 5 mg/kg, and combination treatment, *p* < 0.0001).

The multiple comparisons of withdrawal mean values among the pairs of the seven study groups at 120 min show that the mean values of combination treatment and pregabalin (30 mg/kg) are significantly higher than the mean values of the five groups (sham control, CCI control, etanercept at 40 mg/kg, etanercept at 20 mg/kg, and pregabalin at 5 mg/kg, *p* < 0.0001), with no significant difference between the mean values of the combination treatment and pregabalin (30 mg/kg) groups, *p* = 0.441. Among the mean values of the pairs of these five groups, the mean values of the sham control and pregabalin (5 mg/kg) are significantly higher than the mean values of the other three groups (CCI control, etanercept at 40 mg/kg, and etanercept at 20 mg/kg), with no significant difference between the mean values of the sham control and pregabalin at 5 mg/kg (*p* = 0.094). Among the mean values of the pairs of these three groups (CCI control, etanercept at 40 mg/kg, and etanercept at 20 mg/kg), no statistically significant difference was observed. The multiple comparisons of withdrawal mean values among the pairs of the seven study groups at 180 min show that the mean values of the combination treatment, pregabalin (30 mg/kg), and sham control groups are significantly higher than the mean values of the four groups (CCI control, etanercept at 40 mg/kg, etanercept at 20 mg/kg, and pregabalin at 5 mg/kg), with no significant difference between the mean values of the pairs of combination treatment, pregabalin (30 mg/kg), and sham control groups. Among the mean values of the pairs of the other four groups, the mean values of pregabalin (5 mg/kg) and etanercept (40 mg/kg) are significantly higher than the mean values of the other two groups (CCI control and etanercept at 20 mg/kg), with no significant difference between the mean values of pregabalin at 5 mg/kg and etanercept at 40 mg/kg, and no significant difference between the values of the CCI control and etanercept at 20 mg/kg groups.

### 3.2. Co-Administration of Low Doses of Etanercept and Pregabalin Attenuated Thermal Hyperalgesia in CCI Mice

**Figure 3:**
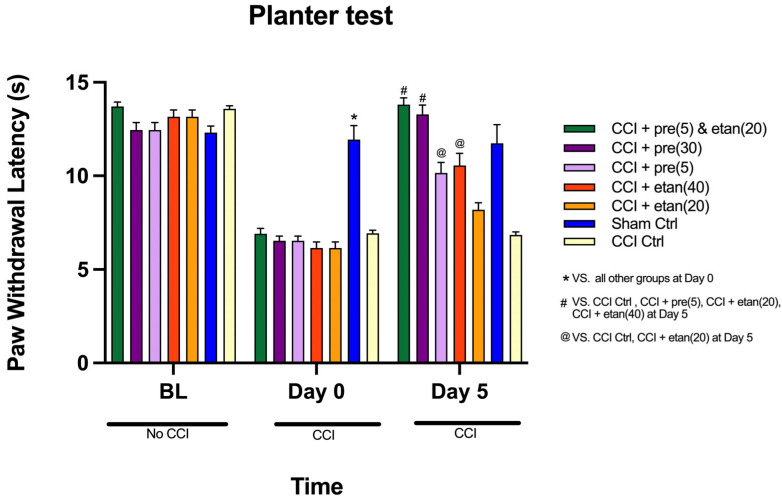
The comparison of mean values of paw withdrawal latency (in seconds) at three time points (baseline, day 0, and day 5) among seven groups: sham control, CCI control, pregabalin at 5 mg/kg, pregabalin at 30 mg/kg, etanercept at 20 mg/kg, etanercept at 40 mg/kg, and combination treatment. All drug treatments were administered intraperitoneally (i.p.). The data are expressed as the mean ± SD of eight mice, and the two-way ANOVA measurement followed by Tukey’s post hoc test was used to determine statistical significance. * Statistically significant (*p* < 0.05) for the sham control group compared to all other groups at day 0. # Statistically significant (*p* < 0.05) for combination treatment and pregabalin at 30 mg/kg compared to CCI control, pregabalin at 5 mg/kg, etanercept at 20 mg/kg, and etanercept at 40 mg/kg at day 5. @ Statistically significant (*p* < 0.05) for pregabalin at 5 mg/kg and etanercept at 40 mg/kg compared to CCI control and etanercept at 20 mg/kg at day 5.

The mean values of paw withdrawal latency (in seconds) across each of the three time points (baseline, day 0, and day 5) among the seven groups in the planter test were compared using a two-way repeated measures analysis of variance (Figure 3). The analysis indicates a statistically significant difference in the mean values of paw withdrawal latency across the three points [F (1, 42) = 470.87; *p* < 0.0001]. Moreover, the interaction of time points and the groups show a highly statistically significant difference in the mean values of paw withdrawal latency [F (1, 8) = 19.56; *p* < 0.0001]. Figure 3 illustrates the comparison of paw withdrawal latency mean values across the seven groups at three time points (baseline, 0-time point, and day 5). At baseline, there was no statistically significant difference between the six groups, suggesting that all groups started with similar pain thresholds. However, at the 0-time point, a highly significant difference emerged between the sham control group (CCI control) and the other five groups (*p* < 0.0001). Figure 3 indicates that the sham control group displayed significantly higher mean PWL values compared to the other groups, indicating greater pain sensitivity post-CCI. There was no statistically significant difference between the other five groups at the 0-time point. By day 5, significant differences in mean PWL values were observed across the groups (*p* < 0.0001). On day 5, the etanercept at 20 mg/kg group showed significantly lower mean PWL values compared to most other groups, except for the CCI control group (*p* < 0.0001). The etanercept at 40 mg/kg group exhibited improved PWL values compared to the etanercept at 20 mg/kg and CCI control groups, but these values were still significantly lower than those of the pregabalin at 30 mg/kg and combination treatment groups (*p* < 0.0001). There was no statistically significant difference between the etanercept at 40 mg/kg and pregabalin at 5 mg/kg groups (*p* = 0.9). The pregabalin at 5 mg/kg group showed modest pain relief, with significantly higher PWL values than the etanercept at 20 mg/kg and CCI control groups (*p* < 0.0001), but the values were still lower than those of the pregabalin at 30 mg/kg and combination treatment groups (Figure 3). There was no significant difference between the pregabalin at 5 mg/kg and etanercept at 40 mg/kg groups (*p* = 0.9), indicating similar efficacy at these doses. By day 5, the pregabalin at 30 mg/kg group exhibited significantly higher mean PWL values compared to the CCI control, etanercept at 20 mg/kg, etanercept at 40 mg/kg, and pregabalin at 5 mg/kg groups (*p* < 0.0001), reflecting strong anti-nociceptive effects. The pregabalin at 30 mg/kg group had no significant difference in mean PWL values compared to the combination treatment group (*p* = 0.96), suggesting that the higher dose of pregabalin alone was as effective as the combined therapy. The combination treatment group, which received low doses of both etanercept and pregabalin, demonstrated the most substantial pain relief by day 5, with PWL values significantly higher than those of the CCI control, etanercept at 20 mg/kg, etanercept at 40 mg/kg, and pregabalin at 5 mg/kg groups (*p* < 0.0001). However, the combination group’s mean PWL values were comparable to those of the pregabalin at 30 mg/kg group (*p* = 0.96).

### 3.3. Impact of Co-Administration of Low Doses of Etanercept and Pregabalin on Thermal Nociceptive Response in CCI Mice 

The comparison of paw withdrawal latency across the three time points (baseline, 0-time point, and day 6) among the seven groups in the hot-plate test revealed highly significant differences across all time points [F (1, 49) = 128.47; *p* < 0.0001]. The interaction between the time points and groups also showed a highly significant difference in mean PWL values [F (1, 8) = 20.107; *p* < 0.0001].

**Figure 4:**
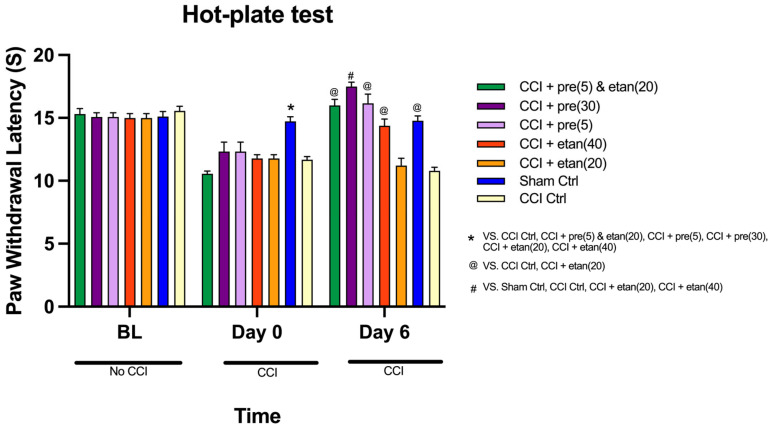
The comparison of mean paw withdrawal latency (in seconds) at three time points (baseline, day 0, and day 6) among seven groups: sham control, CCI control, pregabalin at 5 mg/kg, pregabalin at 30 mg/kg, etanercept at 20 mg/kg, etanercept at 40 mg/kg, and combination treatment of low doses of each drug. All drug treatments were administered intraperitoneally (i.p.). The data are expressed as the mean ± SD of eight mice, and the two-way ANOVA measurement followed by Tukey’s post hoc test was used to determine statistical significance. * Statistically significant (*p* < 0.05) for the sham control group compared to all other groups at day 0. @ Statistically significant (*p* < 0.05) for combination treatment, pregabalin at 5 mg/kg, etanercept at 40 mg/kg, and sham control compared to CCI control and etanercept at 20 mg/kg at day 6. # Statistically significant (*p* < 0.05) for pregabalin at 30 mg/kg compared to sham control, CCI control, etanercept at 20 mg/kg, and etanercept at 40 mg/kg at day 6.

At the baseline, there were no significant differences in PWL among the groups, indicating that all groups started with similar pain thresholds (Figure 4). However, Figure 4 shows that at the 0-time point, the sham control group exhibited significantly higher PWL mean values compared to the other six groups (*p* < 0.0001), reflecting heightened pain sensitivity post-CCI. No statistically significant differences were observed between the other six groups at this time point. By day 6, significant differences in PWL were noted among the groups (*p* < 0.0001). On day 6, the etanercept at 20 mg/kg group showed significantly lower PWL mean values compared to most of the other groups (*p* < 0.0001). The etanercept at 20 mg/kg group had the lowest pain relief among the treated groups, comparable to the CCI control group. The etanercept at 40 mg/kg group exhibited improved PWL values compared to the etanercept at 20 mg/kg and CCI control groups (*p* < 0.0001). However, its mean PWL values were still significantly lower than those of the pregabalin at 30 mg/kg group and the combination treatment group (*p* < 0.0001). There was no significant difference between the mean values of the etanercept at 40 mg/kg group and the combination treatment group (*p* = 0.96). The pregabalin at 5 mg/kg group exhibited moderate pain relief, with significantly higher PWL values than the etanercept at 20 mg/kg and CCI control groups (*p* < 0.0001). However, the pregabalin at 5 mg/kg group did not show significant differences compared to the sham control, etanercept at 40 mg/kg, and combination treatment groups. By day 6, the pregabalin at 30 mg/kg group showed significantly higher mean PWL values than four of the other groups (CCI control, sham control, etanercept at 20 mg/kg, and etanercept at 40 mg/kg; *p* < 0.0001). However, there was no significant difference between the pregabalin at 30 mg/kg group and the combination treatment group (*p* = 0.96). The combination treatment of low doses of pregabalin and etanercept resulted in significantly higher PWL values compared to the CCI control and etanercept at 20 mg/kg groups (*p* < 0.0001). However, the combination treatment showed no significant difference in PWL values compared to the pregabalin at 30 mg/kg and etanercept at 40 mg/kg groups. This suggests that the combination treatment was as effective as the high-dose pregabalin and etanercept at 40 mg/kg (Figure 4).

### 3.4. Effect of Co-Administration of Low Doses of Etanercept and Pregabalin on Locomotor Activity of CCI Mice

The comparison of locomotor counts across three time points (baseline, 0-time point, and day 6) among the seven groups in the locomotor test revealed significant differences over time [F (1, 49) = 77.145; *p* < 0.0001]. Additionally, the interaction between time points and groups showed a significant difference in locomotor counts [F (1, 8) = 9.889; *p* < 0.0001].

**Figure 5:**
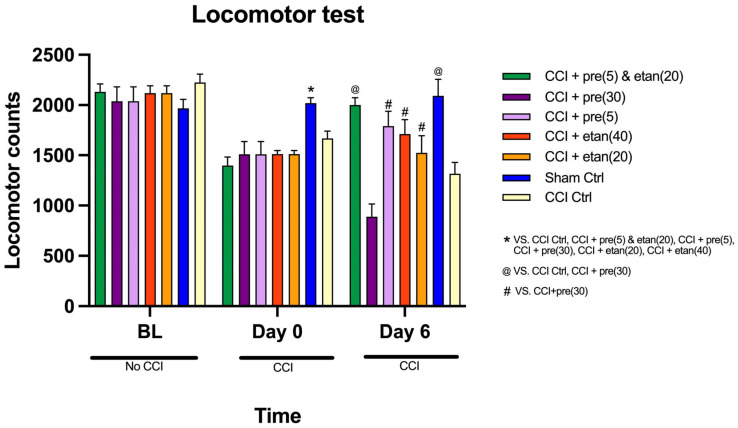
The comparison of mean locomotor counts at three time points (baseline, day 0, and day 6) among seven groups: sham control, CCI control, pregabalin at 5 mg/kg, pregabalin at 30 mg/kg, etanercept at 20 mg/kg, etanercept at 40 mg/kg, and combination treatment of pregabalin and etanercept. All drug treatments were administered intraperitoneally (i.p.). The data are expressed as the mean ± SD of eight mice, and the two-way ANOVA measurement followed by Tukey’s post hoc test was used to determine statistical significance. * Statistically significant (*p* < 0.05) for the sham control group compared to all other groups at day 0. @ Statistically significant (*p* < 0.05) for combination treatment and sham control compared to CCI control and pregabalin at 30 mg/kg. # Statistically significant (*p* < 0.05) for pregabalin at 5 mg/kg, etanercept at 20 mg/kg, and etanercept at 40 mg/kg compared to pregabalin at 30 mg/kg at day 6.

At baseline, no statistically significant differences were found in locomotor counts among the groups (*p* = 0.678), indicating that all groups started at similar levels of activity (Figure 5). However, as shown in Figure 5, at the 0-time point, significant differences were observed across the groups (*p* < 0.0001), with the sham control group showing significantly higher locomotor counts compared to the other groups. By day 6, statistically significant differences persisted among the groups (*p* < 0.0001). At the 0-time point, locomotor counts in the etanercept at 20 mg/kg group were significantly lower than those of the sham control group (*p* < 0.0001), but there was no significant difference when compared to the other treatment groups. By day 6, the locomotor counts of the etanercept at 20 mg/kg group were not significantly different from the sham control group or the other study groups. The etanercept at 40 mg/kg group showed a similar pattern to the low-dose group at the 0-time point (Figure 5), with significantly lower locomotor counts compared to the sham control group (*p* < 0.0001), but no difference from the other treatment groups. On day 6, there were no significant differences between the etanercept at 40 mg/kg group and the other groups, including the combination group. At the 0-time point, the locomotor counts in the pregabalin at 5 mg/kg group were significantly lower than the sham control group but not significantly different from the other groups. Figure 5 shows that by day 6, the locomotor counts of the pregabalin at 5 mg/kg group remained comparable to all other groups, with no significant differences. By day 6, the pregabalin at 30 mg/kg group exhibited significantly lower locomotor counts compared to the other five groups (pregabalin at 5 mg/kg, sham control, etanercept at 20 mg/kg, etanercept at 40 mg/kg, and combination treatment), but no significant difference was found compared to the CCI control group. The combination treatment group displayed locomotor counts that were significantly higher than the CCI control group on day 6 (*p* < 0.0001). However, the combination group showed no significant difference in locomotor counts compared to the other groups (pregabalin at 5 mg/kg, sham control, etanercept at 20 mg/kg, etanercept at 40 mg/kg).

### 3.5. Impact of Co-Administration of Low Doses of Etanercept and Pregabalin on Motor Co-Ordination of CCI Mice

The comparison of latency to fall (in seconds) across the three time points (baseline, 0-time point, and day 5) among the seven groups in the rotarod test showed significant differences over time [F (1, 50) = 1036.04; *p* < 0.0001]. Additionally, the interaction between time points and treatment groups revealed a highly statistically significant difference in latency [F (1, 13) = 61.04; *p* < 0.0001].

**Figure 6:**
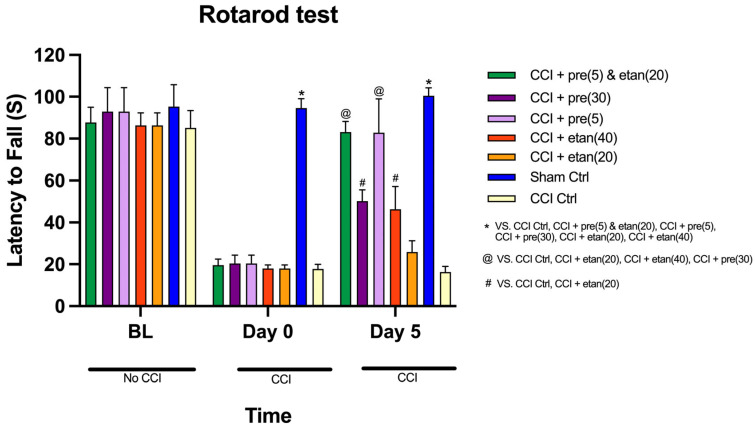
The comparison of mean latency to fall (in seconds) at three time points (baseline, day 0, and day 4) among seven groups: sham control, CCI control, pregabalin at 5 mg/kg, pregabalin at 30 mg/kg, etanercept at 20 mg/kg, etanercept at 40 mg/kg, and combination treatment. All drug treatments were administered intraperitoneally (i.p.). The data are expressed as the mean ± SD of eight mice, and the two-way ANOVA measurement followed by Tukey’s post hoc test was used to determine statistical significance. * Statistically significant (*p* < 0.05) for the sham control group compared to all other groups at day 0 and day 5. @ Statistically significant (*p* < 0.05) for combination treatment and pregabalin at 5 mg/kg compared to CCI control, etanercept at 20 mg/kg, etanercept at 40 mg/kg, and pregabalin at 30 mg/kg at day 5. # Statistically significant (*p* < 0.05) for pregabalin at 30 mg/kg and etanercept at 40 mg/kg compared to CCI control and etanercept at 20 mg/kg at day 5.

At baseline, there was no statistically significant difference among the seven groups (*p* = 0.152), indicating that all groups started at similar performance levels (Figure 6). However, at the 0-time point, significant differences emerged (*p* < 0.0001), with the sham control group displaying significantly higher latency values than all other groups (Figure 6). By day 5, highly significant differences persisted (*p* < 0.0001). Figure 6 illustrates that on day 5, the etanercept at 20 mg/kg group showed significantly lower latency values compared to the etanercept at 40 mg/kg and pregabalin at 30 mg/kg groups (*p* < 0.0001). There was no significant difference between the etanercept at 20 mg/kg group and the CCI control group. The etanercept at 40 mg/kg group exhibited significantly higher latency values than the etanercept at 20 mg/kg and CCI control groups (*p* < 0.0001), reflecting better performance on the rotarod. However, there was no significant difference between the etanercept at 40 mg/kg and pregabalin at 30 mg/kg groups. As seen in Figure 6, on day 5, the pregabalin at 5 mg/kg group showed significantly higher latency values compared to the etanercept at 20 mg/kg and CCI control groups (*p* < 0.0001), but these values were lower than the sham control group. There was no significant difference between the pregabalin at 5 mg/kg and combination treatment groups. The pregabalin at 30 mg/kg group displayed significantly higher latency values compared to the etanercept at 20 mg/kg and CCI control groups (*p* < 0.0001). The pregabalin at 30 mg/kg group had similar latency values to the etanercept at 40 mg/kg group, with no significant difference observed between them. The combination treatment group showed significantly higher latency values compared to the CCI control and etanercept at 20 mg/kg groups (*p* < 0.0001) but no significant difference compared to the pregabalin at 5 mg/kg group (Figure 6). This suggests that the combination treatment achieved similar results as the low dose of pregabalin in improving motor performance. However, the sham control group had significantly higher latency values than both the pregabalin at 5 mg/kg and combination treatment groups (*p* = 0.002).

## 4. Discussion

Previous studies indicate that both pregabalin and TNF-α blockers play potential roles in the management of pain-like behaviors when used in combination, owing to their demonstrated anti-nociceptive effects [15,18,21,55,56].

Behavioral assessments of allodynia and hyperalgesia are widely regarded as essential tools for determining the severity of neuropathic pain [57,58]. This study assessed the anti-nociceptive and anti-allodynic effects by measuring the paw withdrawal latency (PWL), paw withdrawal threshold (PWT), and paw lifts, utilizing the radiant-heat test, von Frey filament test, and hot-plate test, in a corresponding sequence, in CCI mice. The study combined low doses of etanercept and pregabalin, aiming to enhance anti-nociceptive effects through the augmented interaction of two drugs with distinct mechanisms of action. This approach was intended to provide sufficient pain relief at low doses while reducing the intensity and incidence of unwanted side effects, particularly without any discernible effect on motor function and activity. In the present study, significant pain reduction was seen for a high dose of pregabalin in mechanical allodynia and thermal hyperalgesia. Pregabalin, a medication commonly used in the treatment of neuropathic pain, has been widely investigated for its effectiveness and underlying mechanisms. The mechanism of action of pregabalin involves modulating the alpha-2-delta subunit of the voltage-gated calcium channel, leading to analgesic effects [59]. A recent study reported that pregabalin may regulate the P2Y2 receptor through α2δ-1 [6].

Pregabalin is a well-known effective neuropathic pain drug and has proven to be effective in managing conditions such as diabetic neuropathy, post-herpetic neuralgia, and post-spinal cord injury pain [60]. Additionally, studies have emphasized the analgesic properties of pregabalin in different neuropathic pain models, confirming its efficacy in pain relief [15,18,21,56] and in attenuated neuropathic pain [22]. Furthermore, pregabalin has demonstrated efficacy in alleviating neuropathic pain by suppressing the spinal release of glutamate, suggesting a predominant peripheral action in neuropathic pain [61]. However, in our study, a dose of 30 mg/kg of pregabalin demonstrated a significant negative impact on locomotor activity and motor co-ordination as evidenced by the rotarod test results. The mice exhibited drowsiness and sedation, confirming that pregabalin can induce neurological side effects at high doses, affecting both locomotor activity and rotarod test performance. These findings align with those reported by Li et al. (2023), who demonstrated that pregabalin can induce neurological side effects at therapeutic doses, negatively impacting both locomotor activity and rotarod performance [62]. Bannister et al. (2011) further proposed that the analgesic effects of pregabalin are likely mediated through the modulation of spinal neuronal hyperexcitability, which may also account for its impact on locomotor activity [63]. Similarly, in clinical studies, pregabalin is effective in managing neuropathic pain; however, its side effects can impact patient compliance with treatment. Studies have indicated that adverse events such as somnolence, dizziness, and peripheral edema are commonly reported with pregabalin use [64,65].

These findings are promising, given that the use of gabapentinoids is often associated with concerns regarding side effects [66,67]. These side effects are dose-dependent and can lead to the discontinuation of the drug due to intolerability [56]. Because the side effects are dose-related, dose reduction and targeting another neuropathic pain mechanism might add therapeutic value to the treatment of this challenging pain disorder.

Animal studies have shown that administering exogenous TNF-α can induce allodynia in rodents [68,69], whereas TNF-α antagonists have been shown to reduce pain-related behaviors and hyperalgesia in CCI models [28,30]. TNF-α blockers have been found to reduce allodynia in diabetic mice, indicating their potential use in treating diabetic neuropathy [29]. Targeting proinflammatory cytokines represents a promising strategy for managing neuropathic pain, given their pivotal role in chronic pain conditions.

Etanercept, an inhibitor of tumor necrosis factor-alpha (TNF-α), is being explored for its ability to alleviate neuropathic pain by reducing neuroinflammation, a key factor in the development and persistence of neuropathy. The overall results of the tactile allodynia and thermal hyperalgesia tests show that the use of a low dose of etanercept alone does not have any effect on pain attenuation. However, a high dose of etanercept was comparable to a low dose of pregabalin in reducing pain hypersensitivity. This attenuation, however, did not return the hind paw withdrawal response to the normal level. The effects of etanercept on neuropathic pain observed in our study are consistent with previously described findings. Specifically, etanercept has been shown to ameliorate hyperalgesia in animal models of painful neuropathy, such as chronic constriction injury of the sciatic nerve [28]. Additionally, etanercept pretreatment has been reported to delay the onset of mechanical allodynia in a cisplatin-treated mouse model [69]. Furthermore, it was shown that etanercept attenuates thermal and mechanical hyperalgesia in a bone cancer pain mouse model [70]. In an animal model of fibromyalgia, etanercept demonstrated a substantial decrease in pain and decreased pain sensitization in thermal hyperalgesia and mechanical allodynia by lowering the production of pain-related inflammatory mediators, including c-FOS, nerve growth factor (NGF), TNF-α, IL-1β, IL-6, MCP-1, and GFAP. It diminished the activation of microglia and astrocytes [71]. Together, these data suggest that the combination of the two drugs in low doses achieved a similar pain reduction level to the high dose of pregabalin. Our findings conclusively show that a regimen of repeated low doses of etanercept and pregabalin initiated 3 days post-induction during the full establishment of neuropathy and continued for 5 days effectively reversed pain behaviors in the well-established CCI model of neuropathic pain. Specifically, we found that repeated administrations of etanercept at 20 mg/kg and pregabalin at 5 mg/kg were effective in mitigating CCI-induced thermal hyperalgesia and mechanical allodynia in CCI mice. Reducing the doses of pregabalin and etanercept resulted in fewer side effects, as evidenced by the results from the rotarod apparatus and locomotor activity assessments. Prior research has demonstrated that gabapentin and pregabalin can produce synergistic effects when combined with other analgesics in animal studies. In rodent models, pregabalin and gabapentin have been found to enhance pain relief when used in combination with other agents. For instance, both drugs showed improved analgesic effects when administered with tapentadol [72], morphine [73], carbamazepine [74], or tolperisone [22]. In clinical studies, pregabalin was shown to boost the efficacy of duloxetine [75] and oxycodone [76], while gabapentin similarly enhanced the effects of oxycodone [77] and morphine [78]. It has also been noted that naltrexone, an opioid receptor modulator, not only diminishes the nerve injury-induced coupling between µ-opioid receptors and Gs proteins but also enhances oxycodone’s anti-allodynic effects [79].

In the current study, the observed synergistic interaction between etanercept and pregabalin may be attributed to their separate mechanisms of action. The precise mechanism behind their combined anti-allodynic effect remains to be clarified but may arise from their distinct sites and modes of action. Both pregabalin and gabapentin are known to alleviate tactile allodynia by targeting the α2δ-1 and α2δ-2 subunits of L-type voltage-dependent calcium channels [80,81,82]. While pregabalin’s main effect is thought to be the modulation of neurotransmitter release within the spinal cord, some evidence suggests that it may also act within the dorsal root ganglia [61]. Additionally, gabapentin—and likely pregabalin—may reduce glutamatergic signaling in the dorsal horn by indirectly modulating NMDA receptors [83,84,85], and there is also evidence supporting that gabapentin activates potassium channels [86,87]. The primary mode of action may involve indirectly reducing peripheral sensitization rather than directly targeting the central nervous system. Consequently, the augmented analgesic effects observed with the combination of pregabalin and etanercept in this study could result from their simultaneous effects on both peripheral and central pain pathways. The rationale for this combination lies in the distinct mechanisms of action of both agents, each capable of providing analgesia independently while acting at different sites within the peripheral and central pathways of pain transmission.

Future studies should explore the optimal dose ratios of pregabalin and etanercept to maximize the therapeutic benefits while minimizing side effects. Additionally, investigating the impact of both acute and chronic administrations of this combination therapy could provide deeper insights into its mechanism of action. Expanding the analysis to include a broader range of cytokines and their roles in neuropathic pain may further elucidate the potential of this combination therapy. Furthermore, clinical studies are needed to confirm the translational potential of these findings from animal models to patients, particularly in terms of safety and efficacy in managing neuropathic pain. Finally, long-term studies assessing the potential for tolerance and dependence with this combination therapy would be valuable for understanding its clinical applicability. The limitation of this study is that we exclusively utilized male mice. It is well documented that there are sex-dependent variations in the mechanisms that contribute to persistent pain in animal models, and these differences are recognized as significant factors influencing pain behaviors [88,89].

## 5. Conclusions

In conclusion, our findings suggest that combining pregabalin with etanercept may offer therapeutic benefits for alleviating CCI-induced neuropathic pain with minimum side effects, especially in elderly patients who are at higher risk of experiencing adverse effects from pregabalin. This combination may enhance the anti-nociceptive effects, offering advantages such as improved efficacy and the ability to reduce the doses of each drug to mitigate side effects. However, while these potential benefits are promising, further research is warranted to determine the effectiveness of this combination across various pain models and elucidate the possible mechanisms underlying these combination therapies.

## Data Availability

The data supporting this study’s conclusions are obtainable from the corresponding author, L.A., upon request. The data are not publicly accessible owing to privacy and ethical constraints.

## Abbreviations

IASP	International Association for the Study of Pain
Aβ	Type of Peripheral Sensory Fiber (Beta Fibers)
Aδ	Type of Peripheral Sensory Fiber (Delta Fibers)
C fibers	Type of Peripheral Sensory Fiber
DRG	Dorsal Root Ganglion
CNS	Central Nervous System
EPSCs	Excitatory Postsynaptic Currents
NMDA	N-Methyl-D-Aspartate
α2δ-1	Alpha-2/Delta-1 Subunit of Voltage-Gated Calcium Channels
TNF-α	Tumor Necrosis Factor-Alpha
VGSCs	Voltage-Gated Sodium Channels
CCI	Chronic Constriction Injury
PWT	Paw Withdrawal Threshold
PWL	Paw Withdrawal Latency
LA	Locomotor Activity
IL-1β	Interleukin-1 Beta
IL-6	Interleukin-6
IL-17	Interleukin-17
IL-10	Interleukin-10
Ipsi.	Ipsilateral
i.p.	Intraperitoneal
PRE	Pregabalin
ETAN	Etanercept
